# P37 Benchmarking antibiotic use in Community health (CHS), Health and Justice and Mental health services (MHS)

**DOI:** 10.1093/jacamr/dlag102.043

**Published:** 2026-06-26

**Authors:** Naomi Fleming, Hugh Attwood, Harriet Launders, Vanessa Earnshaw, Vivienne Clark, Rachel Medcalf

**Affiliations:** NHS England, UK; NHS England, UK; University Hospital Southampton NHS Foundation Trust, Southampton, UK; Cheshire & Wirral Partnership NHS Foundation Trust, UK; Central London Community Healthcare NHS Trust, London, UK; Nottinghamshire Healthcare NHS Foundation Trust, UK

## Abstract

**Objectives:**

In April 2023 the national Antimicrobial Prescribing and Medicines Optimization (APMO) team, part of NHS England’s Antimicrobial Resistance (AMR) programme, launched a workstream to understand antimicrobial stewardship (AMS) activities and limitations in CHS and MHS including health and justice (HJ) providers. The need for an audit to understand current antibiotic prescribing within these organizations and provide organizations with a benchmark was identified and a working group set up to: (i) design and develop a benchmarking audit tool; (ii) promote the audit tool and gather data across organizations; (iii) collate the data to allow benchmarking between like organizations; and (iv) identify areas for AMS improvement.

**Methods:**

A working group developed 3 audit standards and a simple benchmarking audit tool for use in CHS, HJ and MHS. The initial tool was piloted, refined based on feedback and then launched nationally with an agreed data collection period. All live antibiotic prescriptions by all routes, including those from third parties, were audited for one week during two weeks in October 2025. Data was collated, analysed and results shared with organizations for use during World Antimicrobial Resistance Awareness Week in November 2025. Organizations were categorized depending on the services they provide.

**Results:**

28 organizations participated in the audit. Half the organizations provided both CHS and MHS and submitted 83% of the audit data (1211) prescriptions. These organizations significantly influenced the overall audit results. Organizations providing only CHS had the lowest response rate submitting only 29 audited prescriptions across 2 organizations. 1453 prescriptions were audited. 92% were compliant with NICE and local guidelines or Microbiology advice. 88% were prescribed with the correct course length. 15% were broad-spectrum antibiotics (65% co-amoxiclav, 21% cefalexin, 10% ciprofloxacin, 2% levofloxacin and 1% ceftriaxone). HJ prescriptions (*n*=74) showed the highest compliance with antibiotic guidelines for both choice (99%) and course length (91%) and lowest percentage of broad-spectrum antibiotics (9%). MHS prescriptions (*n*=139) had the lowest compliance with antibiotic guidelines for choice (82%). CHS prescriptions (*n*=29) had the lowest compliance with antibiotic guidelines for course length (79%) and highest broad-spectrum use (24%). Overall, flucloxacillin was the most prescribed antibiotic (*n*=227), followed by nitrofurantoin (*n*=202) and doxycycline (*n*=198). Metronidazole (*n*=29) scored the lowest percentage for correct antibiotic choice (76%). Broad spectrum use was driven by co-amoxiclav (*n*=147). 88% prescriptions were correct choice but only 68% had the correct course length.

**Conclusions:**

The audit standards and tool were appropriate for use in CHS, HJ and MHS; multiple organizations participated. The collation of data and split by organization type enables organizations to benchmark their results against their peers. Most antibiotic choices aligned with guidance, however, some incorrect use of metronidazole and co-amoxiclav, suggests education on correct use of these antibiotics is required. Course lengths were correct in 88% of prescriptions; this continues to be an opportunity for improvement. Next steps include updating the tool for Access Watch and Reserve category data collection, differentiating prescribing by third-party organizations and encouraging wider adoption of the audit next year.

